# Assessing the psychometric properties of the Chinese return-to-work self-efficacy questionnaire using Rasch model analysis

**DOI:** 10.1186/s12955-022-01929-7

**Published:** 2022-02-16

**Authors:** Feng Liu, Zhenxiang Zhang, Beilei Lin, Zhiguang Ping, Yongxia Mei

**Affiliations:** School of Nursing and Health, Zhengzhou University, No. 100 Science Avenue, Zhengzhou, 450001 Henan China

**Keywords:** Stroke, Return-to-work, Self-efficacy, Psychometrics, Rasch analysis

## Abstract

**Background:**

Self-efficacy is a significant predictor of return to work and affects the confidence of survivors to return to work after illness. The Return-to-work self-efficacy (RTW-SE) questionnaire is a self-report questionnaire to assess confidence in returning to work with good reliability and validity. The aim of this study was to translate and cross-culturally adapt the RTW-SE questionnaire into Chinese and examine the psychometric properties among young and middle-aged stroke survivors using Rasch model analysis.

**Methods:**

The cross-cultural adaptation and translation procedures followed a dual-translation approach. The psychometric properties of the RTW-SE questionnaire were examined using Rasch model analysis by Winsteps software. The unidimensionality and local independence were analyzed by principal component analysis of the residuals (PCAR) and standardized residual correlations.Category diagnostics were performed for scale function, and the item fit, reliability, and separation were also validated. Item-person maps were used to examine the distribution and matching of item’s location and person ability. Finally, the differential item functioning (DIF) was used to measure gender-related group equivalence.

**Results:**

A total of 366 participants aged 23–59 years were recruited from three communities in Zhengzhou. The RTW-SE questionnaire demonstrated unidimensionality and a 5-point Likert rating scale was more appropriate to investigate young and middle-aged stroke survivors’self-efficacy. There was a good fit for the items with both person and item reliabilities greater than 0.8 and separation indices of 3.75 and 3.94, respectively. The item location was identified from the item-person map as not covering person ability, but the scale did not have an age-related DIF.

**Conclusions:**

The results confirm evidence of appropriate psychometric properties of the RTW-SE questionnaire and can be used as a reliable and validated instrument for measuring self-efficacy to return to work in young and middle-aged Chinese patients with stroke.

## Introduction

Stroke is a leading cause of mortality and disability worldwide and the economic costs of post-stroke care are substantial. Results form the Global Burden of Disease Study (GBD) showed that stroke was the second most common cause of disability-adjusted life years as well as the second largest cause of deatth after ischaemic heart disease, and the highest age-standardised incidences of stroke were observed in China [1]. There is a concerning shift in the overall stroke burden toward younger age groups with a gradual increase in the proportion of young and middle-aged stroke survivors [2, 3]. Stroke causes various degrees of dysfunction, involving activities, language, swallowing, and cognition, These make the daily activities of survivors are restricted and work participation is hindered [4].

Return to work was defined as people who leave their work due to injury or illness and then re-engage in the original work, return to a similar work or start a new work, including paid part-time and full-time employment [5]. The inability to return to work has adverse effects on quality of life and leads to loss of economic productivity [6, 7], and return to work is an important sign of recovery and return to normal life. A variety of factors influence the return to work of stroke survivors,, involving individuals, families, society and other aspects. Among them, the self-efficacy plays an important role in the return to work [8, 9].

In the return to work process, self-efficacy has usually been described as three broad categories according to the functional domain: general self-efficacy [10, 11], job self-efficacy [12], and return-to-work self-efficacy (RTW-SE) [13, 14]. RTW-SE is the employee's belief that they can meet the requirements required to return to work [15]. In contrast to work self-efficacy, which emphasizes that the employees are already at work, RTW-SE emphasizes the process of returning to work for employees [16] and is an important predictor of return to work [8, 17]. The results of literature indicate that RTW-SE has a facilitative effect on stroke survivors in the early stages of return to work [18]. Higher RTW-SE shortens the time to return to work [19] and facilitates early return to work and maintains working status [20]. Therefore, the increase in RTW-SE of patients facilitates their return to normal social life, which leads to physical and psychological recovery, reduction of family financial burden and improvement of quality of life. In conclusion, the assessment of RTW-SE in stroke survivors is crucial to the vocational rehabilitation process.

Several methods have currently been developed to measure RTW-SE; however, limitations of these measures already exist. The Return-To-Work Self-Efficacy Scale (RTWSE) developed by Brouwer and the 19-item Return-To-Work Self-Efficacy Scale developed by Shaw are specifically designed for the work-injured population [21, 22]. The Job Procurement Self-Efficacy Scale (JPSE) was designed by Wenzel to focus on the healthy population, and the 11-item Return-To-Work Self-Efficacy Scale developed by Black focuses only on patients after returning to work [23, 24]. These instruments are designed to a single symptom and are not applicable to stroke patients with more complex residual dysfunction. In contrast, the Return-to-Work Self-Efficacy Questionnaire has clear and explicit scoring criteria that have been shown to be useful for people with mental illness, musculoskeletal disorders, cancer patients and other people. It has application value and practical significance in stroke population. The RTW-SE questionnaire was developed by Lagerveld et al. [13] in 2010 to measure the level of RTW-SE in survivors after illness. Participants are asked to respond to statements related to their work and imagine that they would return to full-time work tomorrow. Questionnaire development through interviews with stakeholders (e.g. clinical psychologists, work and organizational psychologists, occupational physicians and workers with health problems) and informing them of the purpose of the scale assessment provides care providers with useful information to deliver tailored interventions. The questionnaire can be used after full return to work or during the return to work process. Because of its predictive value, the RTW-SE questionnaire can be used as a screening tool in clinical practice or vocational settings to provide direction for return to work after illness, in addition, caregivers can design more rational interventions based on RTW-SE scores.

China has a significant trend towards younger stroke survivors, with only 17% and 11% of its urban and rural stroke survivors returning to work within one year post discharge, respectively [25], which causes a greater socioeconomic loss. The literature suggests that increasing RTW-SE is effective in reducing the time to return to work for stroke survivors [26], facilitating their early return to work, and is a significant predictor of their return to work status [27]. Therefore, in order to fulfill the survivor's desire to return to work as soon as possible, it is necessary to measure their confidence in returning to work in order to improve the rehabilitation program and develop targeted interventions, which can be achieved with the RTW-SE questionnaire. The purpose of this study was therefore to translate, culturally adapt, and validate a Chinese version of the RTW-SE so as to provide support for its initial application among young and middle-aged stroke survivors. Thus, we postulated the following: (1) the RTW-SE questionnaire was adapted for application to stroke patients in a Chinese cultural context;and (2) the RTW-SE questionnaire has appropriate psychometric properties.

## Methods

### Translation and cross‑cultural adaptation

After obtaining the authorization of professor Lagerveld from the original scale development team, this study follows Brislin's model of translation and cross-cultural adaptation, through a forward and backward translation approach in the following steps [28]. First of all, the original English version of the questionnaire was translated into Chinese by two native Chinese bilingual translators, A1 (a bilingual doctor of nursing with study abroad experience) and A2 (a teacher majoring in English), who were the two researchers. Then the two translated versions of the questionnaire were compared with the original scale by researcher C, whose native language is Chinese, and the subject research team conducted a discussion to determine the final Chinese direct translation tone and version. Secondly, the back-translation was done by two other bilingual researchers, B1 (a nursing instructor with a stroke background) and B2 (a professional English instructor with a medical background), who had not been exposed to the original questionnaire, independently translated the Chinese version of the scale into English. The English back-translated version was obtained by comparing the differences between the back-translated version and the original version by the research team and the translators. And next, the revised Chinese direct translation and English back translation versions were integrated and sent to the original scale developers, who determined whether the language translations were appropriate and consistent. In addition, a team of experts related to the research area of this topic (three experts in neurorehabilitation, two experts in stroke care, one expert in cerebrovascular disease treatment, and one expert in psychology) was invited by mail, and the members of the expert team independently reviewed the original RTW-SE questionnaire, the direct translation version, and the back translation version based on their professional theoretical knowledge and practical experience. Finally, five cases of middle-aged and young stroke patients were finally selected for cognitive interviews and 30 patients were pilot testinged to obtain feedback and understanding, and the Chinese version of the RTW-SE questionnaire was finally developed after modification by comments from various parties.

### Participants

A convenience sampling method was used to select young and middle-aged stroke patients in the three communities in Zhengzhou City, Henan Province from august 2020 to April 2021 as the study population. Inclusion criteria were (1) age between 18 and 59 years; (2) current status of sickness, early retirement, or unemployment; (3) stable condition, normal cognition, and no significant communication impairment; and (4) voluntary participation in this study. Exclusion criteria were patients who were participating in other studies or/and had concurrent major diseases such as cardiac failure, respiratory failure, malignancy, severe trauma, and other critical illnesses. All participants were given full information about the study and all signed an informed written consent form. The sample size should be 5–10 times the number of scale items according to the reliability and validity test [29] and 366 stroke patients were included in this study, taking into account the missing sample size and 10% sampling error.

### Data collection

Before the survey, five members of the research team were trained to introduce the survey method, content interpretation and scoring criteria. During the survey, the participants were guaranteed to fully understand and answer the questionnaire according to their own situation, and the questionnaire was collected on the spot. Participants were asked to complete a socio-demographic form, the modified Rankin Scale (mRS) and the Chinese version of the RTW-SE questionnaire.

### Measures

#### Demographics

The socio-demographic form included items related to gender, age, type of stroke and current employment status. The mRS assesses the recovery of neurological function after stroke and to reflect the degree of disability or dependence of the patient in daily life. The scale has a total score of 6, with higher scores indicating more severe disability. The participants in this study were all young and middle-aged stroke survivors, and patients with an mRS score of 5–6 (total disability or death) should be excluded if they are to be able to return to work.

#### RTW-SE questionaire

The Chinese version of the RTW-SE questionnaire measures the confidence of young and middle-aged stroke patients to return to work. RTW-SE questionnaire consists of 11 items (Table 1), including the questions "I won’t be able to complete my work tasks", "I will be able to perform my tasks at work", "I will be able to concentrate on my work ". Each item is scored using a 6 point scale (1 = strongly disagree, 5 = strongly agree). The mean score of all items was used to represents the scale score, with score above 4.5 indicates a high sense of self-efficacy to return to work. The scale consists of one factor and has good internal consistency reliability, with Cronbach's α coefficients of 0.90 to 0.96. the RTW-SE predicted return to work status (not returned, partially returned, or fully returned) three months after illness, with good predictive reliability.

**Table 1:** Items of the Chinese Version of the RTW-SE questionaire

Ítems	x ± sd
1. I will be able to cope with setbacks	4.27 ± 1.275
2. I won't be able to complete my work tasks due to my emotional state	3.91 ± 1.435
3. I will be able to set my personal boundaries at work	4.19 ± 1.179
4. I will be able to perform my tasks at work	4.23 ± 1.350
5. I will be able to deal with emotionally demanding situations	4.34 ± 1.116
6. I will have no energy left to do anything else	4.09 ± 1.356
7. I will be able to concentrate on my work	4.22 ± 1.245
8. I will be able to cope with work pressure	4.20 ± 1.270
9. I won't be able to handle potential problems at work	4.02 ± 1.320
10. I can motivate myself to perform my job	4.44 ± 1.093
11. I can deal with the physical demands of my work	3.90 ± 1.477

### Statistical analysis

Data item and analysis statistical analysis were performed using IBM SPSS Statistics 21. Descriptive statistics were performed on participant demographics and questionnaire results. Quantitative information (mRS and RTW-SE questionnaire results) was expressed as mean and standard deviation. Qualitative information (demographic information) was expressed as frequency. All Rasch model analysis were performed using the software of Winsteps (version 3.72). Rasch analysis and validation of participant responses and survey items were performed using the Winsteps ' partial credit model.

### Unidimensionality and local independence

Unidimensionality of the items was assessed using a principal component analysis of the residuals (PCAR) [30]. The questionnaire can be considered to satisfy the unidimensionality requirement when the eigenvalue of the first residual contrast is less than 3.0 and the percentage of variance explained by the first contrast is 5% or less [30, 31]. Local independence of items is an important prerequisite for all inferences made by item response theory. The standardized residual correlations from the Rasch model analysis were used to verify that if the residual correlation (r < 0.3) indicated the presence of local independence.

### Item characteristic curve

The item characteristic curve (ICC) is used to describe the relationship between the test response probability and the level of the intrinsic trait, and is defined as An ogive-shaped plot of the probabilities of a correct response to an item for any value of the underlying trait in a respondent [32].

### Category description

In the Rasch model, the intersection of two adjacent category probability curves is the threshold of the item category. The function of the RTW-SE questionnaire was analyzed to assess the suitability of the Liker 6 point for the scale items. The evaluation criteria are as follows: (1) regular observation distribution (e.g. normal, bimodal, slightly skewed distribution, etc.) and a minimum of 10 observed count each category; (2) average measure increased as the category increased; (3) the outfit mean square was less than 2.0 in each category; (4) the category threshold varies monotonically, generally increasing by at least 1.4 logit to show the difference between the two categories, but not exceeding 5 logit to avoid excessive spacing between variable classes [33, 34].

### Item-person map

This “item–person” map or “variable” map is often called a “Wright map “, which places the estimated value measured by the sample respondents and the average location of all items on the same common scale (logits) [32]. The estimation of the item is called the item location which refers to the location of the item on the logit scale; the evaluation of the person is called the person’s ability, and it informs each person’s rank in the same common scale. A higher value indicates a more higher location or a more capable person [35]. The item-person map shows the distribution and relative position of individual return-to-work self-efficacy levels of RTW-SE questionnaire.

### Item fit

The Rasch model assesses the fit of the observed data to the Rasch model by comparing the degree of difference between the theoretical probabilities of the subjects' test responses and the actual observed data. Infit and Outfit statistics which indicate the information-weighted mean square residuals between observed and expected responses are usually used as statistical indicators for items fit tests. Mean-quare (MnSq) is the mean-square infit or outfit statistic with expectation 1. Values substantially less than 1 indicate dependency in data and substantially greater than 1 indicate noise. Infit and Outfit MnSq less than 0.6 indicates an overfit item, greater than 1.4 indicates an underfit item [36]. The point-measure correlation has a range of − 1 to + 1, indicating how close the item is to its measurement target, with higher correlation coefficients indicating that the item is closer to the measurement target.

### Reliability and separation

The reliability and separation of person and items were examined. Reliability refers to the reproducibility of person and item measures, separation means dividing people's ability or item location into different levels, reflecting the number of different levels that the sample can be divided into [32]. Reliability greater than 0.8 indicates good repeatability of the test, separation greater than 2 indicates that the test has sufficient discrimination for people or items [37].

### Differential item functioning (DIF)

Differential item functioning (DIF) is a type of differential validity that refers to differences in the performance of different subgroups of individuals with underlying traits on the same item, i.e., the statistical properties of the item differ for different subgroups of individuals [38]. DIF was used to analyze the probability of understanding and responding to the item by individuals of different genders, and if the response probability was higher for both females than males, it showed that the item had DIF in terms of gender. DIF contrast indicates the difference between the DIF logit measures of each subgroup. The significance level of DIF was set to 0.05, and items were considered biased when Mantel–Haenszel (M–H) DIF size > 0.64 logits, p < 0.05 [39].

## Results

### Translation and adaptation of RTW-SE

The English back-translated version of the scale is very consistent with the original version, and the original author states that "finish" refers to the completion of a task, which is different from the concept of "perform". Translators and experts tinkered with the wording of some statements in the scale in the Chinese cultural context, and then modified the scale scales in the context of the interviews and pre-experiments. The final translation of scale 3 is "I will be able to work within my personal scope of work or ability". Adding the prerequisite of being in the working state to entries 5 and 6, the final translation resulted in item 5, "I will be able to handle emotionally stressful situations at work," and item 6, "I will not have the energy to do anything else but work.

### Sample characteristics

The 366 participants (248 males and 118 females) who finally completed the study had a mean (50.70 ± 7.12) years of age ranging from 23 to 59 years, more than half of the participants were mainly manual laborers (46.8%). Up to 88.5% of the participants are haemorrhagic stroke, mostly with varying degrees of residual functional impairment. The highest number of participants had an mRS score of 1 (57.9%), and roughly the same proportion of participants were on sick leave (51.2%) or unemployed (41.5%). The details can be seen in Table 2.

**Table 2:** Demographic and medical characteristics (n = 366)

Variable	n (%)
Gender	
Male	248(67.8)
Female	118(32.2)
Age (years)	
Mean (SD), range	50.70(7.12),23–59
Type of job	
Intellectual labour	93(24.6)
Manual labour	171(46.8)
A mixture of both	101(27.6)
Current employment status	
Sick leave	202(52.2)
Early retirement	11(3.0)
Unemployed	152(41.5)
Type of Stroke	
Heamorrhagic	37(10.1)
Ischaemic	324(88.5)
Mixed	5(1.4)
mRS score	
0–1	259(70.7)
2–4	107(29.2)

*SD* standard deviation*, mRS* modified Rankin Scale

### Unidimensionality and local independence

The RTW-SE questionnaire met the unidimensionality and local independence requirements of the Rasch model. The analysis of dimensionality found variance explained by the measure was 65.3%, and eigenvalue of the first contrast was 1.7 with an associated unexplained variance of 5.5%. The residual correlation coefficients for item of the questionnaire ranged from -0.30 to -0.21, and the absolute values were all less than 0.3, so local independence of this questionnaire was established.

### Item characteristic curve

**Fig. 1:**
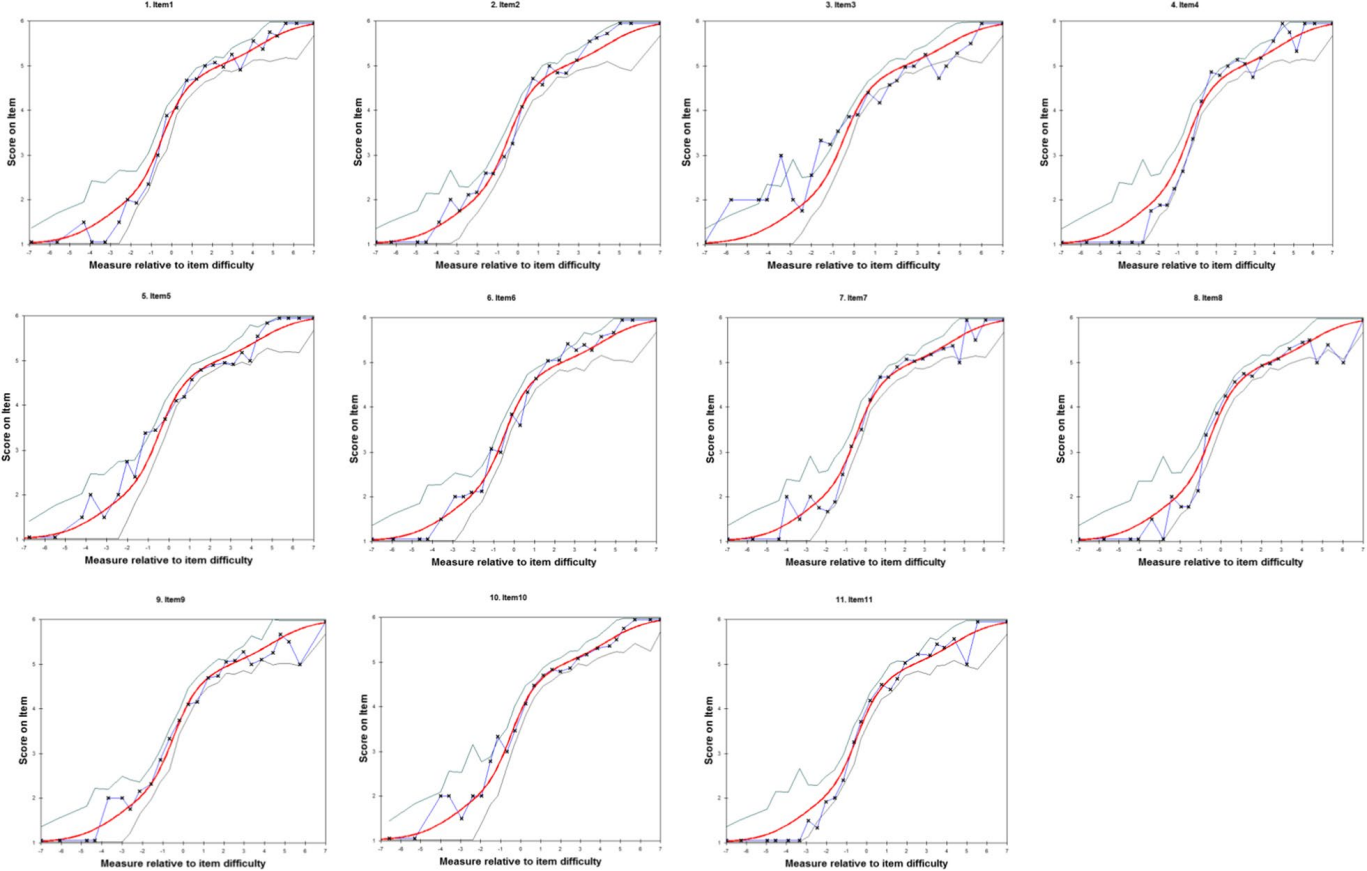
Item characteristic curves of RTW-SE questionnaire

Figure 1 shows the ICC of the RTW-SE questionnaire. The red line is the item characteristic curve as expected by the Rasch model and the blue line is the empirical ICC, “X” are the means of the measures and ratings for observations in the interval. When the " X " on the blue line is at or very close to the red line, the test is a good fit to the model. The green-gray lines are two-sided 95% confidence bands. These are 1.96 standard errors vertically away from the red line. We can see based on Fig. 1, the empirical ICC curves fit well with the expected ICC curves for most of the items, except for item 3. The category for Item 3 at locations − 3.4, 1.2, 4, and 4.5 failures to fit expected and exceed the 95% confidence interval.

### Category description

Table 3 shows the summary of rating scale function. It can be seen that the observation distribution of category lable in 6-point Likert questionaire has a bimodal regular distribution and the number of observations is greater than 10, its average measurement value increases monotonically as the category labels are sorted, and the mean square of Outfit for the six categories is in the range of 0.72 to 1.98, which are within the acceptable range. However, the category thresholds did not increase monotonically, and the threshold for category 4 was smaller than that of category 3, indicating that the number of response categories needs to be reconsidered.

**Table 3:** Suitability of RTW-SE questionaire

Likert-type	Category	Observed Counts	Average measure	Outfit MnSq	Category thresholds
6-point	1	123	− 2.68	1.98	None
	2	544	− 1.18	1.42	− 3.46
	3	390	− 0.52	0.72	− 0.45
	4	806	0.34	0.72	− 0.73
	5	1815	1.80	1.00	0.20
	6	348	3.49	1.00	4.45
5-point	1	123	− 3.34	1.61	None
	2	544	− 1.56	1.21	− 4.08
	3	1196	− 0.01	0.74	− 1.52
	4	1815	2.18	0.93	0.64
	5	348	4.06	1.10	4.96

Since there was no monotonic increase in the threshold for category 4, category 4 was considered to be combined with either category 3 or category 5. If category 4 was combined with category 5, more than half of the observations were in one category, so the choice was made to integrate category 4 with category 3. The results showed that the observed distribution was regular and each category was selected more than 10 times, the mean measures were ranked according to the category labels, and the mean square of Outfit for each category was less than 2. Also the category thresholds were monotonically increasing and the difference between adjacent categories was more than 1.4 logit and less than 5 logit. It can be concluded that the RTW-SE questionnaire with a 5-point Liker scale is more appropriate.

### Item-person map

**Fig. 2:**
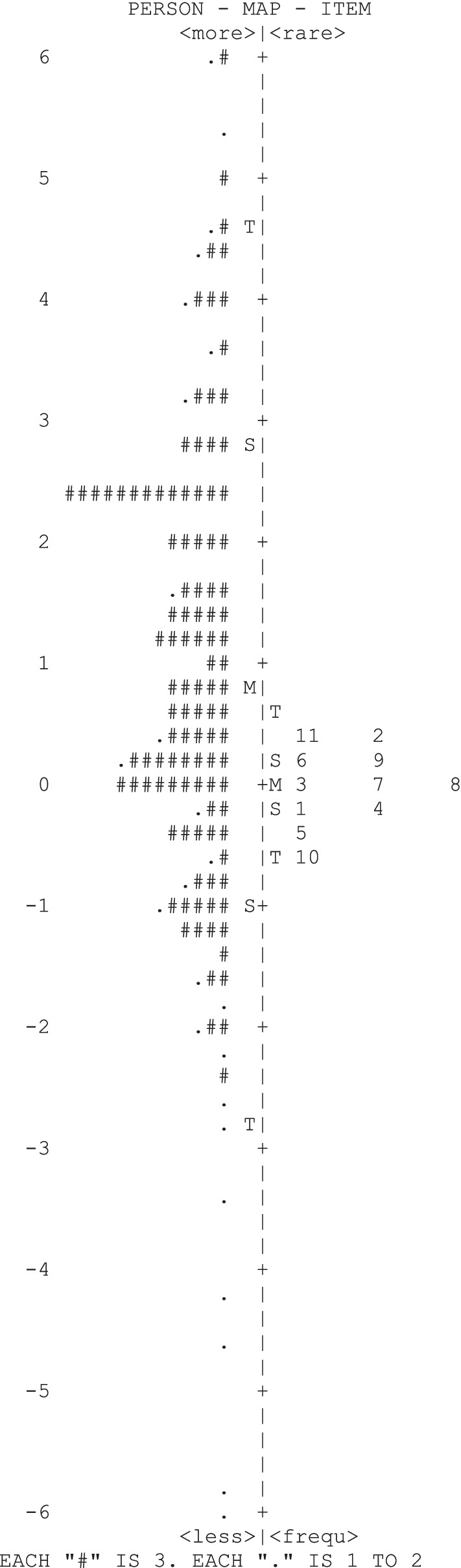
Item-person map of RTW-SE questionaire

Figure 2 shows the item-person map of RTW-SE. The left side of the figure shows the ability of the patients, with "#" and "." indicates the location of the distribution of the person measure, with the level of RTW-SE of patients decreasing in order from top to bottom. Similarly, the right side of the figure depicts the items in order of location level, with the highest location at the top (2, 11) and the lowest location at the bottom (10). As shown in the figure, the levels of RTW-SE in young and middle-aged stroke patients are widely distributed, but the location of items were slightly biased towards medium stroke patients, and there is a lack of items for patients with higher and lower ability levels in this group. This indicates that it is less accurate to measure stroke patients at both ends of the ability scale, and therefore more items with location differences should be developed to address patients with different abilities, so as to improving the scale's discrimination.

### Item fit

The RTW-SE item location, standard errors (SE), and associated Infit and Outfit statistics are shown in Table 4. Higher Logits scores indicated that patients have higher levels of RTW-SE. The RTW-SE item location was estimated to be between -0.50 and 0.45 logits. Question 11(I can deal with the physical demands of my work)position was the highest, with a score of 0.45 logit (SE = 0.07). Question 10 (I can motivate myself to perform my job)position was the lowest, with a score of -0. 50 logit (SE = 0.07). The infit statistic for item 3(infit = 1.64; outfit = 1.83) did not fit the requirements. After removing it, the MnSq values of the remaining items were in the range of 0.6–1.4 and fit the Rasch model well, which can be used to evaluate the self-efficacy of young and middle-aged stroke patients during the return to work process. All items of the questionnaire had PT-measure correlations greater than 0.6, indicating that all items functioned in the same direction to predict latent trait. In addition, the standard errors (S.E.) were all 0.07, and the statistics were stable with the model fit.

**Table 4:** Item analysis statistics

Item	Measure	SE	Infit MnSq	Outfit MnSq	PT-measure
1	− 0.17	0.07	0.77	0.76	0.78
2	0.43	0.07	1.22	1.35	0.76
3	− 0.03	0.07	1.64^a^	1.83^a^	0.62
4	− 0.11	0.07	0.72	0.84	0.80
5	− 0.32	0.07	1.00	1.02	0.75
6	0.14	0.07	1.11	1.06	0.77
7	− 0.10	0.07	0.66	0.66	0.79
8	− 0.06	0.07	0.74	0.76	0.77
9	0.25	0.07	1.11	1.14	0.74
10	− 0.50	0.07	0.78	0.83	0.76
11	0.45	0.07	1.06	1.11	0.78

*SE* standard errors, *MnSq* mean-square

^a^Item misfit (infit index > 1.4)

### Reliability and separation

The Rasch analysis resulted in a person reliability of 0.93 and a separation of 3.75, showing good levels of confidence and separation to distinguish between approximately four levels of person ability. Item reliability was 0.94 and separation was 3.94, indicating that the sample size was sufficient to confirm the ranking of items on the RTW-SE continuum.

### Differential item functioning (DIF)

Analysis of the DIF based on the RTW-SE questionnaire for gender in young and middle-aged stroke patients showed that M-H size of the uniform DIF item ranged from − 0.62 to 0.52 (*p* > 0.05). For non-uniform DIF, there were 4 positive items that disadvantaged male low-ability patients. In contrast, there were 2 positive items that disadvantaged female high-ability patients and 4 where they had an advantage over male high-ability subgroup. Overall, there were more items that disadvantaged Macau students but all items were not significantly different. This indicates that men and women had the same location in answering the questionnaire items and that there was no DIF for the RTW-SE questionnaire items with respect to gender.

## Discussion

In this study, the RTW-SE questionnaire was translated and cross-culturally adapted, and the Rasch rating scale model was used to validate the structure of the scale to investigate its applicability to the young and middle-aged stroke population.

The Rasch model is an advanced measurement theory that overcomes the limitations of some measures of the traditional Likert scale based on classical test theory [33]. Rasch analysis allows for in-depth validation of scale items and potentially proves the best quality criteria for the measurement [40]. The questionnaire items basically satisfied the unidimensionality assumption statistically thought the application of the Rasch rating scale model. After applying the Rasch rating scale model, it was found that the existing category of items consisting of a 6-point Likert scale was not suitable for young and middle-aged stroke survivors. The category probability curve revealed that category 4 had higher thresholds than category 3 which violated the principle of monotonically increasing category thresholds. Therefore, category 3 "slowly disagree" and category 4 "slowly agree" are combined into one category (recorded as "neutral "), the reconstructed 5-point Likert scale meets the functional requirements of the scale and might be more appropriate for measuring RTW-SE in stroke survivors who are unsure of their confidence in returning to work.

The Item-person map from the RTW-SE questionnaire reveals a significant limitation and mismatch between item location level and person ability level. The location of the questionnaire items is higher than -0.50 and lower than 0.45 while the human ability level is between -7.07 and 8.05, which shows that the location of the items is cannot meet the human ability level. In addition, nearly half of the items were sorted by location with a neighboring location difference of less than 0.06, indicating that most respondents would answer these items in a similar manner. The overall location of the items was lower than the mean value of person ability, which is applicable to moderate levels of self-efficacy and cannot fully cover low and high levels of people. Therefore, easier or more difficult items need to be added to improve the differentiation of the items in order to apply the questionnaire effectively to people with lower or higher levels of ability.

The conformity analysis of the RTW-SE questionnaire items showed no problems within the statistical range of adaptation. In addition, good reliability and separation were reported for all items and persons. No biased items were found in this study when comparing the potential ability levels of male and female subjects which shows that there is no DIF with respect to gender. Therefore, this cross-cultural adaptation study on the RTW-SE questionnaire not only reveals the applicability of the tool in the Chinese context but also finds new evidence for the validity and reliability of the tool.

Returning to work which is often used as an outcome measure in work and health prediction and intervention studies is an important concept in the field of vocational rehabilitation [41]. A study about Canadian stroke survivors reported that young and middle-aged stroke patients had a strong desire to return to work but had less confidence because of their perceived ability to work was reduced and the functional impairment caused by stroke [42]. Young and middle-aged stroke survivors expect to return to work but were hindered by physical disability and impaired image after the disease because they fear discrimination by social groups and loss of confidence in returning to work. Therefore, the questionnaire can be used for vocational rehabilitation of young and middle-aged stroke survivors in China. On the one hand, it can provide a basis for accurately assessing the confidence of patients who are leaving or unemployed due to illness or returning to work after illness, and helps medical staff to more clearly understand the impact of illness on patients' confidence in returning to work. On the other hand, based on the important predictive value of RTW-SE for returning to work makes understanding patients' confidence and exploring its main influences can provide reference for the construction of return to work intervention programs.

The study has some limitations. First, most of the study population included in this study were young and middle-aged people about 3 months after stroke and only baseline idata was collected from the patients, limiting the generalizability of the findings. Second, there is a lack of ongoing follow-up of study participants to explore the predictive value of RTW-SE size on when to return to work. Third, the patients participating in this study are currently out of work lacking exploration of those who have returned to work. The study of stroke survivors in different work states would provide a comprehensive understanding of the psychological characteristics of this population and allows for different measures to facilitate return to work. Fourth, Rasch analysis was unable to assess discriminative ability across different levels of subjects, so future research could use other methods of item response theory to provide this information. Fifth, the nature of the RTW-SE (state/trait) will affect the reliability and validity of the measurement instrument, which may affect the results of this study [43]. This issue should be clarified in future studies. Finally, in order to facilitate the clinical application of this questionnaire, the minimum clinical important difference of the instrument should perhaps be further evaluated [44].

## Conclusion

A RTW-SE questionaire was validated in the Rasch analysis with a sample of Chinese patients with middle-aged stroke. It is recommended to use the 5-point Likert scale instead of the category level of the original scale to validate the RTW-SE levels in Chinese stroke patients. Although the presence of significant floor and ceiling effects necessitated additional modifications, this instrument could support healthcare practitioners in developing targeted intervention strategies by studying the levels of RTW-SE in patients.

## Data Availability

The datasets generated and analysed during the current study are not publicly available due the fact that they constitute an excerpt of research in progress but are available from the corresponding author on reasonable request.

## Abbreviations

DIF: Differential item functioning
M-H: Mantel-Haenszel
MnSq: Mean-quare
mRS: Modified Rankin Scale
RTW-SE: Return-to-work self-efficacy
SE: Standard errors
SD: Standard deviation
